# Na^+^/K^+^-pump and neurotransmitter membrane receptors

**DOI:** 10.1007/s10158-018-0221-7

**Published:** 2018-11-28

**Authors:** Arkady S. Pivovarov, Fernando Calahorro, Robert J. Walker

**Affiliations:** 1 Department of Higher Nervous Activity, Faculty of Biology, Moscow Lomonosov State University, Moscow, Russia 119234; 2 Biological Sciences, Faculty of Environmental and Life Sciences, University of Southampton, Life Sciences Building 85, Southampton, SO17 1BJ UK

**Keywords:** Na^+^/K^+^-pump, Ouabain, Neurotransmitter membrane receptors

## Abstract

Na^+^/K^+^-pump is an electrogenic transmembrane ATPase located in the outer plasma membrane of cells. The Na^+^/K^+^-ATPase pumps 3 sodium ions out of cells while pumping 2 potassium ions into cells. Both cations move against their concentration gradients. This enzyme’s electrogenic nature means that it has a chronic role in stabilizing the resting membrane potential of the cell, in regulating the cell volume and in the signal transduction of the cell. This review will mainly consider the role of the Na^+^/K^+^-pump in neurons, with an emphasis on its role in modulating neurotransmitter receptor. Most of the literature on the modulation of neurotransmitter receptors refers to the situation in the mammalian nervous system, but the position is likely to be similar in most, if not all, invertebrate nervous systems.

## Introduction

**Fig. 1:**
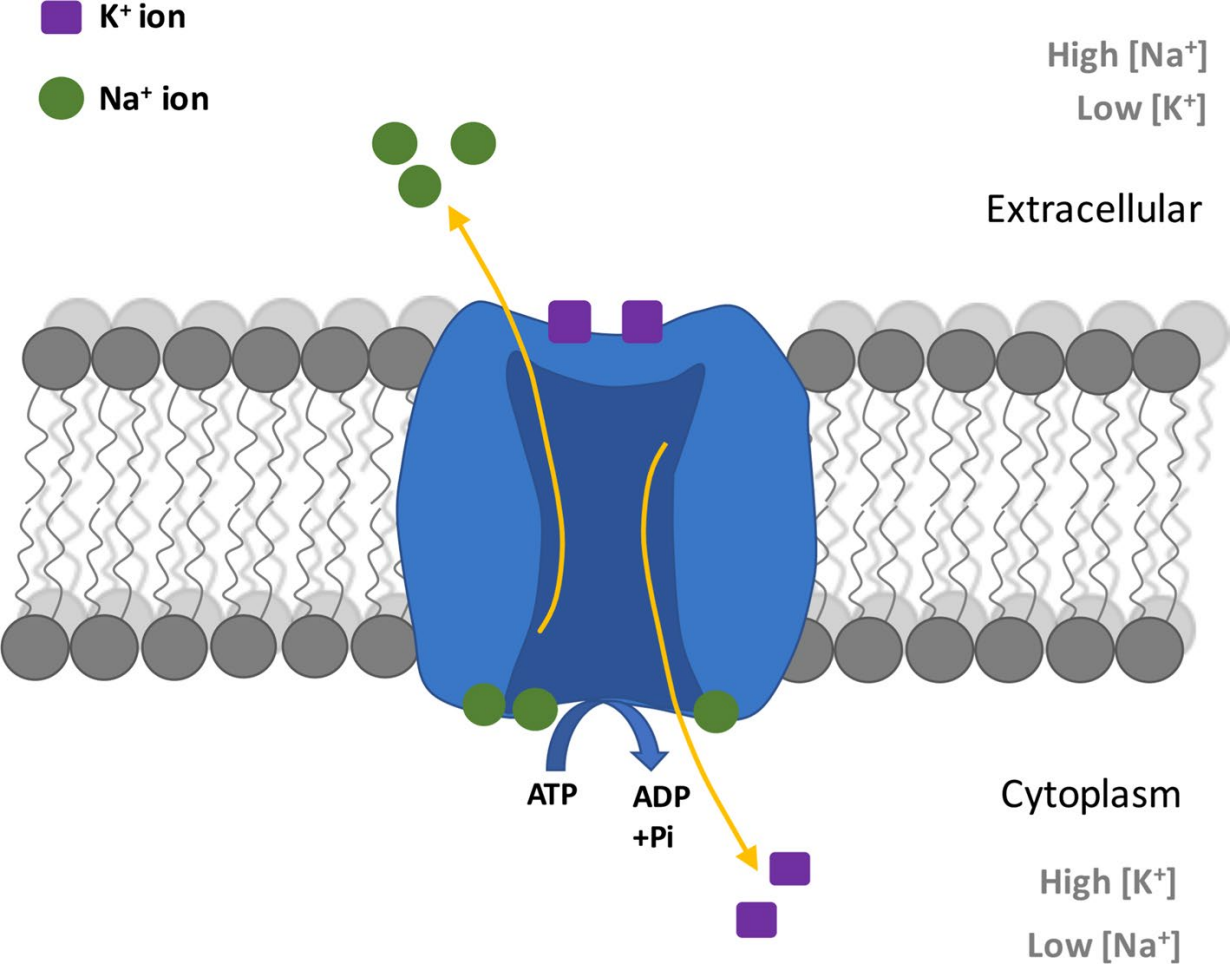
A diagram showing the extrusion of 3 Na^+^ from the cell while 2 K^+^ entering the cell due to the activation of the Na^+^/K^+^-ATPase enzyme. This results in a raised concentration of intracellular K^+^ and a reduced concentration of intracellular Na^+^ relative to the interstitial fluid

There is an ionic imbalance between the inside and outside of a cell, with K^+^ concentration high inside the cell and low outside, while the Na^+^ concentration is low inside the cell and high outside. The cell membrane is differentially permeable to K^+^ and Na^+^, being more permeable to K^+^ than to Na^+^ in the resting state. Thus, there is a continuous tendency to loose K^+^. To maintain this ionic gradient, the cell membrane has a Na^+^/K^+^-pump which requires a source of energy, viz, ATP. However, this pump, the Na^+^/K^+^-ATPase protein, is not electroneutral since for each 3 Na^+^ removed from the cell, 2 K^+^ enter which requires one ATP (Thomas 1972b). This is summarized in Fig. 1. The importance of this function is reflected in estimates that suggest it accounts for roughly 50% of the total brain energy consumed (Erecinska and Silver 1994). This action of the pump results in the cell losing net positive charge, becoming more negative, resulting in a hyperpolarization of the cell membrane potential. As a result, the Na^+^/K^+^-pump is termed electrogenic, contributing to the resting membrane potential in probably all cells, including striated muscle, cardiac muscle, vascular muscle, enteric muscle, salivary and other glands and neurons. A Mg^2+^-activated Na^+^/K^+^-ATPase was first described in nerves from the crab, *Carcinus maenas* (Skou 1957, 1989), and reviewed by Skou and Esmann (1992). The evolutionary history of the Na^+^/K^+^-pump has been investigated in depth by Saez et al. (2009), and the Na^+^ pump occurs in all metazoan phyla, including Cnidaria, for example, in *Hydra vulgaris* (Canfield et al. 1992). The difference in concentration of monovalent cations created by this enzyme is associated with key reactions of living cells, namely generation of excitation, cell osmotic regulation/cell volume, ionic homeostasis, cell cycle regulation and the regulation of cellular metabolism (Boldyrev 1993). Boldyrev also reviews the situation when ionic pumps operate under pathological conditions. There is also a role for Na^+^/K^+^-ATPase in the regulation of endosomal pH (Cain et al. 1989; Fuchs et al. 1989).

## Molecular features

**Fig. 2:**
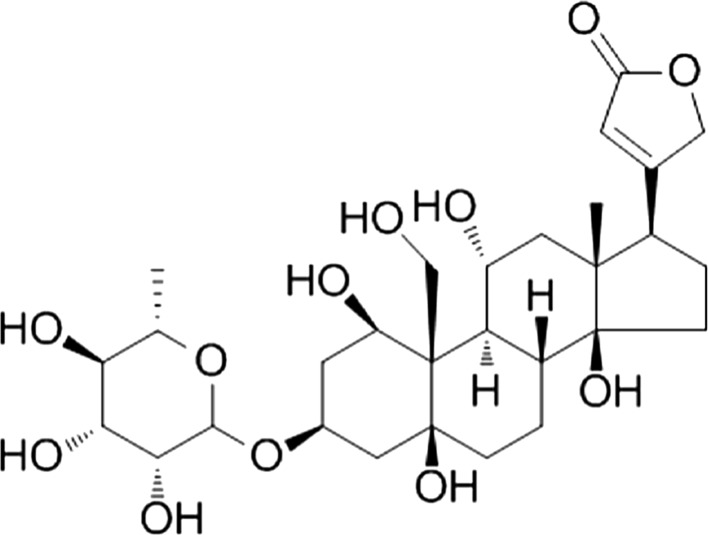
Structure of the cardiac glycoside, ouabain, a specific inhibitor of Na^+^/K^+^-ATPase

Na^+^/K^+^-ATPase can be considered as an enzyme, ATPase, or an ion transporter, Na^+^-pump, but these are two aspects of the same function. The Na^+^/K^+^-pump is composed of three subunits, viz, *α*, *β* and *γ* (Kaplan 2002; Li and Langhans 2015). The large catalytic *α* subunit, a protein of ~ 110 kDa, is responsible for the transport activity of the enzyme and has an ATP binding site and phosphorylation site. The extracellular domain and transition region possess binding sites for the cardiac glycoside, ouabain (Fig. 2), a specific inhibitor of Na^+^/K^+^-ATPase. The regulatory single-transmembrane-domain *β* subunit, a protein of ~ 55 kDa, is a highly glycosylated transmembrane protein which increases the translation efficiency and stability of the *α* subunit. In mammals, there are three *α* and two *β* subunits with *α*3 and *β*2 being expressed mainly in neurons and *α*2 and *β*1 being expressed mainly in glia. *α*1 is expressed ubiquitously. The *α* and *β* subunits can assemble to form functional Na^+^/K^+^-ATPase, but there are also examples of subunits existing on their own (Okamura et al. 2002). The *γ* subunit, a FXYD protein, is more tissue specific and modifies the affinity for Na^+^, K^+^ and ATP, pump kinetics and transport properties and further stabilizes Na^+^/K^+^-ATPase. The *α* and *β* subunits belong to a large superfamily of evolutionary conserved transmembrane ATPases, called P-type as they have a phosphorylated intermediate during the reaction cycle. *α* subunits have been identified in all invertebrate phyla (Saez et al. (2009), and a complete list of P-type ATPase genes in *Caenorhabditis elegans* and *Drosophila melanogaster* can be found in Okamura et al. (2002). There are 21 P-type ATPase *α* subunits and three *β* subunits in *C. elegans*, while in *D. melanogaster* there are 15 *α* and six *β* subunits.

## Role in neurons

The Na^+^/K^+^-pump is an active transporter that uses ATP hydrolysis as an energy source to move both ions across the neuronal membrane against their concentration gradients and has specific functions associated with the generation of the action potential, as well as with the maintenance of other active transport mechanisms, regulation of the cell volume (Karpova et al. 2010a, b) and heat generation (Clarke et al. 2013). This current review is primarily concerned with studies on the Na^+^/K^+^-pump in neurons and its role in the regulation of transmitter receptors but will first provide an overview of its cellular functions. However, the reader will see that work on its role in the regulation of transmitter receptors has primarily be undertaken on mammals, but this work indicates the way for future research using invertebrate neuronal preparations.

## Na^+^/K^+^-pump and membrane potential

The membrane potential (MP) can be divided into a diffusion potential, determined by the relative permeability of the membrane for Na^+^ and K^+^ and an additional potential arising from the operation of an electrogenic Na^+^ pump. Under normal conditions of the Na^+^/K^+^-pump, the MP is approximately 10 mV more electronegative than if it were due only to passive ion fluxes. This highlights the important contribution played by the active transport mediated by the transporter. Thomas (1972a, 1982), in experiments on *Helix aspersa* (now classified as *Cornu aspersum*, but in this review the name used in the literature quoted will be used) giant neurons, found that injection of Na^+^ hyperpolarized these neurons by up to 25 mV and this hyperpolarization was prevented in K^+^-free saline. He showed that ouabain reduces MP in these neurons by around 3 mV, reaching a constant value after 6 min. The same time is necessary for the inhibition of the Na^+^/K^+^-pump with ouabain, whose reaction speed was determined by the change in the intracellular activity of Na^+^, measured by the Na^+^ selective microelectrode. [Na^+^]_*i*_ was calculated to be 3.6 mM but increased in the presence of ouabain and when external K^+^ was reduced to zero. The maximum depolarization of *Helix pomatia* neurons caused by ouabain was about 7 mV (Christoffersen 1972). However, the size of the depolarization due to cardiac glycosides depends on the neuron under investigation. Lambert et al. (1974) found two types of neuron in *H. aspersa*, one depolarized by strophanthidin and a second type that was resistant. The average depolarization to strophanthidin was 8.2 mV with little or no change in membrane resistance. These authors concluded that strophanthidin-sensitive neurons were relatively permeable to sodium and the coupling between Na^+^ and K^+^ was calculated as 3Na^+^:2K^+^. Na^+^/K^+^ pump makes a significant contribution to the MP of the mollusc neurons because of the high specific resistance of their membranes (about 1 MΩ/sm^2^) (Kononenko 1976). To maintain a balance between the pump and passive membrane currents, many more Na^+^/K^+^-pump molecules are required, approximately 1000 times more than channel proteins for K^+^ and Na^+^ (Daut 1987). The distance between Na^+^/K^+^-pump molecules is, on average, only 34 nm. Thus, the membrane has a high density of pump molecules. The fact that the flow of Na^+^ ions into the cell and K^+^ ions from the cell is compensated by the pump has another consequence, which is to maintain a stable osmotic pressure and constant volume (Daut 1987).

The Na^+^/K^+^-pump also plays a role in regulating the rhythmic activity of giant neurons of *Helix* by activating their inter-burst hyperpolarization (Ayrapetyan 1973). During neuron depolarization, the intracellular Na^+^ concentration rises sufficiently high to activate the pump. This results in the MP becoming more negative and a reduction in pacemaker neuron activity. This effect is blocked by 1 mM strophanthidin-K and 0.2 mM dinitrophenol. When the neuron is silent, strophanthidin-K depolarizes the cell, but in potassium-free saline, strophanthidin-K has no effect. Raising the temperature also stimulates the MP of pacemaker neurons to become more negative, and this effect increases following intracellular injection of sodium ions. This effect of temperature is dependent on external potassium. There have been many studies demonstrating a role for the electrogenic Na^+^/K^+^-pump in regulating bursting activity of mammalian neurons. For example, blocking the Na^+^/K^+^-pump with strophanthidin (30 µM) or zero potassium increases the spontaneous firing rate and rate of the MP becomes more positive in rat suprachiasmatic nucleus neurons (Wang and Huang 2006). In addition, Strophanthidin reduced the peak after-hyperpolarization (AFP), while zero potassium increased the AFP peak.

The role of the Na^+^/K^+^-pump in regulating bursting activity in oscillator heart interneurons which pace the central pattern generator of the heart of the leech, *Hirudo medicinalis*, has been studied (Kueh et al. 2016). These authors stimulated the Na^+^/K^+^-pump of the oscillator heart interneurons by increasing intracellular Na^+^, using either an intracellular Na^+^ electrode or the Na^+^/H^+^ antiporter, monensin. Raising intracellular Na^+^ results in stimulating an outward current that hyperpolarizes the neurons. This hyperpolarizing current is blocked with strophanthidin (100 µM), indicating the current is associated with activation of the Na^+^/K^+^-pump. This hyperpolarizing current in turn activates an *h*-current that depolarizes the MP and enhances bursting of the oscillator neurons. Leech sensory neurons also have a Na^+^/K^+^-pump that induces a prolonged hyperpolarization following a burst of activity that is blocked by strophanthidin, 500 µM (Jansen and Nicholls 1973).

## Na^+^/K^+^-pump and regulation of cell volume

The important role of the Na^+^/K^+^-pump in the regulation of cell volume is supported by the fact that when animal cells are treated with ouabain, inhibiting the Na^+^/K^+^-pump, they swell and burst. Under normal conditions, a major role in preventing cell rupture due to osmotic pressure belongs to the Na^+^/K^+^-pump (Alvarez-Leefmans et al. 1992). There is a correlation between the activity of the Na^+^/K^+^-pump and the volume of the cell (Ayrapetyan 1990). Activation of Na^+^/K^+^-pump leads to a decrease in cell volume, and inactivation of the pump to its increase due to an increase in intracellular Na^+^ concentration. Conversely, an increase in tonicity, when the cell shrinks, inhibits the activity of the Na^+^/K^+^-pump, while the swelling of the cell causes stimulation of the activity of the pump (Suleimanyan et al. 1984; Ayrapetyan 1990; Alvarez-Leefmans et al. 1992). The negative correlation between the activity of the Na^+^/K^+^-pump and the cell volume is based on the change in the number of functionally active pumping molecules in the membrane. A minimum number of Na^+^/K^+^-ATPase molecules are active at rest. Partial inhibition of the electrogenic Na^+^/K^+^-pump causes swelling of the cell and an increase in the number of functioning Na^+^/K^+^-ATPase molecules in the membrane. This compensates for the effect caused by the inhibition of the pump. Activating the pump causes the cell to decrease in size and close some of the functioning Na^+^/K^+^-ATPase molecules, thus preventing unnecessary energy consumption (Suleimanyan 1990). However, this effect of Na^+^/K^+^ pump inhibition on cell volume has been challenged for *H. aspersa* neurons (Alvarez-Leefmans et al. 1992). These authors monitored neuron cell volume and MP in the presence of ouabain and found that while a few neurons briefly swelled and then shrank in the presence of ouabain, most neurons only shrank. In four of 20 neurons, there was no volume change. In all cases, ouabain caused the MP to become more positive. Alvarez-Leefmans et al. (1992) concluded that while the Na^+^/K^+^-pump may be required for long-term cell volume maintenance, neurons have ouabain-insensitive mechanisms which prevent swelling and lysis following pump inhibition.

The mechanism underlying ouabain-induced swelling of the leech, *H. medicinalis*, Retzius cells have been investigated using triple-barrelled ion-sensitive electrodes (Dierkes et al. 2006). Ouabain (500 µM) increased intracellular Na^+^ concentration, decreased intracellular K^+^ concentration, increased intracellular Cl^−^ concentration and depolarized the MP. The ouabain-induced swelling was due to a net uptake of NaCl. In the absence of either extracellular Na^+^ or Cl^−^, the cell volume did not change while in the absence of extracellular K^+^, the Na^+^/K^+^-pump was inhibited, and cell swelling did not occur. The authors concluded that the ouabain-induced NaCl uptake, resulting from the MP becoming more positive, stimulated Cl^−^ entry down its electrochemical gradient.

## Na^+^/K^+^-pump and astrocytes

The Na^+^/K^+^-pump in astrocytes is required to maintain a low K^+^ level around neurons (Wang et al. 2012). It mediates an increase in K^+^ uptake which results in a transient decrease in extracellular K^+^ levels around neurons. Activation of Na^+^/K^+^-ATPase is mediated by Ca^2+^ partly through Na^+^ influx via a Na^+^/Ca^2+^ exchanger.

## Na^+^/K^+^-pump and intracellular ion composition

The most significant physiological impact of the cardiac glycoside, ouabain (Fig. 2), is related to its effect on the concentration of intracellular Ca^2+^ (Blaustein 1993). Inhibition of the Na^+^/K^+^-pump by ouabain increases the level of Ca^2+^ in neurons (Fujino and Fujino 1982; Deitmer et al. 1987; Meyer-Lehnert et al. 2000). The inhibition of the pump increases the level of Na^+^ in the cytoplasm; this activates the Na^+^/Ca^2+^ exchange in the reverse mode. The increased concentration of Ca^2+^ causes Ca^2+^ release from the intracellular Ca^2+^ depot, which evokes further increases in the level of this cation in the cytoplasm (Balduini and Costa 1990; Condrescu et al. 1995; Saghian et al. 1996; Rakovic et al. 1999). Although there is evidence that low concentrations (< 1 µM) of ouabain act directly on the Na^+^/Ca^2+^ exchange to increase intracellular Ca^2+^ (Saghian et al. 1996). Following inhibition of the Na^+^ pump with ouabain, the intracellular concentration of Na^+^ and ATP increases, which activates the reversed Na^+^/Ca^2+^ exchanger and increases intracellular Ca^2+^ concentration from the extracellular medium (DiPolo and Beaug 1986; Deitmer et al. 1987; Ayrapetyan 1990; Schlue 1991; Mark et al. 1995; Saghian et al. 1996; Pivovarov and Boguslavskii 2001). Another way to increase intracellular Ca^2+^ with ouabain is to mobilize deposited Ca^2+^ by releasing it from intracellular Ca^2+^ stores through activation of ryanodine and IP_3_ receptors (Micci and Christensen 1998; Calvino et al. 2002).

An increase in the concentration of free Ca^2+^ in the dialysed neurons of the pond snail (*Lymnaea stagnalis*) and grape snail (*H. pomatia*) reduces the amplitude of the inward Cl^−^ current caused by acetylcholine (ACh) (Chemeris et al. 1982; Arvanov et al. 1992b). An investigation of the inhibitory effect of ouabain on the somatic cholinergic receptors of the neurons of the grape snail has shown that the amplitude of the ACh current depends on the concentration of free Ca^2+^ in the cytoplasm (Pivovarov and Boguslavskii 2001).

## Endogenous ouabain-like compounds

There have been a number of candidates for endogenous digitalis-like factors and ouabain-like compounds in animal tissues, including in the nervous system (Goto et al. 1992; Bagrov and Shapiro 2008), which can affect brain function. For example, block of an endogenous ouabain-like compound in the rat locus coeruleus reduces rapid eye movement sleep, an event associated with expression of Na^+^/K^+^-ATPase (Jaiswal et al. 2010). An endogenous ouabain-like compound has been identified in human plasma (Hamlyn et al. 1991) which is structurally, biologically, and immunologically indistinguishable from ouabain.

## Na^+^/K^+^-pump and auditory system

The Na^+^/K^+^-pump is involved in the maintenance of the osmotic balance in the mammalian inner ear (Bartolami et al. 2011; Lang et al. 2007). The vestibular endolymph that bathes the sensory hair cells resembles intracellular fluid with a high concentration of K^+^ (150 mM), low Na^+^ (2 mM), Ca^2+^ (20 µM) and a positive potential of + 80 mV (Hibino et al. 2010). When the steriocilia of the hair cells are activated, K^+^ enters the cells and depolarizes them and these in turn excite the neurons they innervate. A number of ion channels and transporters expressed in the inner ear, including Na^+^/K^+^-ATPase expressed in the stria vascularis, are involved in endolymph homeostasis.

The role of the Na^+^/K^+^-pump in auditory mechanosensation has been investigated in *D. melanogaster* (Roy et al. 2013). These authors investigated the expression of *α* and *β* subunits of Na^+^/K^+^-ATPase in Johnston’s organ, a chordotonal organ, responsible for hearing in the fly. There are three *α* subunits, viz, ATP*α*, *α*-like (CG3701) and JYalpha, and three *β* subunits, viz, Nrv1 (nervana 1), Nrv2 (nervana 2) and Nrv3 (nervana 3) in the fly. When ATP*α* but not *α*-like (CG3701) is knocked down in the entire chordotonal organ or only in the scolopale cells, which enwrap the organ’s neuronal dendrites in endolymph-like compartments, the insect becomes deaf. ATP*α* has a high expression in the scolopale cells. When the *β* subunit, Nrv2, is knocked down, ATP*α* expression is also reduced, resulting in almost complete deafness. It is proposed that only *nrv2* gene has a scolopale cell-specific expression. *nrv3*, which is only expressed in chordotonal neurons, is also required for hearing in *D. melanogaster*. *nrv1* is expressed at low levels in cap cells. Roy et al. (2013) propose that the Na^+^/K^+^-pump in the fly auditory system is present in the abluminal plasma membrane of the scolopale cells where it actively transports K^+^ into the scolopale cells. K^+^ then passes to the receptor lymph to maintain a high K^+^ concentration, similar to the generation of endolymph in the scala media of the vertebrate inner ear.

Defects in Na^+^/K^+^-ATPase in *D. melanogaster* mutants have been reported to induce a bang-sensitive-like phenotype following mechanical shock (Schubiger et al. 1994). A defective Na^+^/K^+^-ATPase may cause hypersensitivity and a bang-sensitive response. These flies are more sensitive to ouabain compared to wild type. Bang-sensitive behaviour can be induced in wild type by injecting sublethal doses of ouabain.

## Na^+^/K^+^-pump and the visual system

*D. melanogaster* visual system expresses one type of *α* subunit, ATP*α*, and three *β* subunits, Nrv1-3 (Baumann et al. 2010). Loss of Na^+^/K^+^-ATPase *α* subunit, ATP*α*, in photoreceptors of *D. melanogaster* eliminated light-induced depolarization of the photoreceptors, resulting in blindness (Luan et al. 2014). Intracellular recordings from photoreceptors of flies where ATP*α* was knocked down showed they were depolarized in the dark, due to loss of intracellular K^+^. ATP*α* knockdown also caused degeneration of photoreceptors in older flies. Loss of Nrv3, a Na^+^/K^+^-ATPase *β* subunit, partially reproduced the signalling and degenerative defects seen with ATP*α* knockdown. These results show that loss of Na^+^/K^+^-pump activity results in both loss of visual function and induces age-related degeneration of fly photoreceptors. There is also evidence that in the optic lobe of *D. melanogaster* the levels of *α* subunit (ATP*α*) of Na^+^/K^+^-pump oscillate during the day and night (Gorska-Andrzejak et al. 2009). This rhythm is controlled by a circadian clock since it is absent in the arrhythmic mutant, *per*^*0*^. These results indicate that the expression of ATP*α* is affected by light. Changes in the levels of the *β* subunit, Nrv2, also showed a daily rhythm, but these changes were not statistically significant.

## Na^+^/K^+^-pump and activity of neurotransmitter receptors

The Na^+^/K^+^-pump affects neurotransmitter receptors, viz, their density and sensitivity to their transmitter, and these effects will now be considered.

*ACh receptors* Mammalian receptors for ACh are divided into nicotinic and muscarinic receptors. These receptors can be further divided into subtypes, viz, M1–M5 muscarinic receptors (Caulfield and Birdsall 1998) and 16 nicotinic receptor subtypes, viz, *α*1–*α*9, *β*1–*β*4, one *γ*, one *δ*, one *ε* (Lukas et al. 1999). In molluscs, three ACh receptors were initially functionally identified in *Aplysia*, as a rapid excitatory response receptor, a rapid inhibitory response receptor and a slow inhibitory response receptor (Kehoe 1972). The two rapid response receptors are nicotinic, but the slow inhibitory response receptor has unique properties and is activated only by ACh, carbamylcholine and arecoline. Pinsker and Kandel (1969) proposed that a cholinergic *Aplysia* interneuron, L10, activated a follower neuron, not through a change in membrane conductance, but through activation of an electrogenic Na^+^/K^+^-pump. However, it was shown that at least part of this postsynaptic response was due to an increase in K^+^ permeability (Kehoe and Ascher 1970). In 1980, Arvanov and Ayrapetyan published an article about the depressive effect of ouabain on the amplitude of the ACh-induced current of *Helix* neurons. This was an important observation and initiated studies on the regulation of the activity of neurotransmitter systems by the Na^+^/K^+^ pump.

Since fractions of endogenous ouabain-like compounds, endobains, have been found in the mammalian brain (Rodriguez De Lores Arnaiz et al. 1998), it is impossible to exclude the possibility that these compounds also occur in invertebrates and continuously regulate transmitter receptors through changes in the activity of the pump in invertebrate nervous systems. An increase in the level of endobain can suppress the activity of the pump and so reduce the facilitating effect of Na^+^/K^+^-ATPase on the activity of the cholinergic system which could also be the case for invertebrates. For detailed information on the evolution of endogenous ouabain-Na^+^/K^+^ pump interactions, the reader is referred to the excellent review by Blaustein (2018).

In a subsequent more detailed paper (Ayrapetyan et al. 1985), the correlation between Na^+^/K^+^ pump activity and membrane chemosensitivity was analysed using intracellular dialysis of *Helix* neurons. The effects on ACh and GABA-induced membrane currents and ^3^H-*α*-bungarotoxin (^3^H-*α*-BT) and ^3^H-GABA binding in *Helix* ganglia were analysed following changes in pump activity and intracellular ATP. Exposure to either extracellular 100 µM ouabain, or a potassium-free solution, suppressed the ACh-induced current in A-type dialysed neurons. An increase in the intracellular level of ATP led to a depression of the ACh current and the disappearance of the blocking effect of ouabain on these currents. Intracellular ADP had a similar but less significant effect on the currents caused by ACh, while intracellular AMP was ineffective. This effect of intracellular ATP on ACh current was suppressed by dinitrophenol, an inhibitor of membrane phosphorylation. Ayrapetyan et al. (1985) propose that membrane phosphorylation decreases the affinity of membrane receptors for ACh and GABA.

The binding of ^3^H-*α*-BT and 3H-GABA to membranes was inhibited by both ouabain-containing and potassium-free solutions, and by theophylline and NaF, that both increase the levels of intracellular ATP. These results show that the Na^+^/K^+^-pump modulates the affinity of membrane receptors for ACh and GABA. The fact that this was similar to effects seen by modulators of phosphorylation suggests the effects of pump activity are mediated by the phosphorylated state of their receptors.

In a later paper, Arvanov et al. (1992b) showed that ouabain selectively suppressed *Helix* A-type neuron responses to ACh, which were due to the selective increase in membrane permeability to chloride. This effect of ouabain is mediated by an increase in cAMP levels. In contrast, responses of *Helix* B-type neuron, caused mainly by an increase in monovalent cationic permeability, were unaffected by ouabain. The blockade of Cl^−^ responses was not associated with a change in the reversal potential of the response. Arvanov et al. (1992a) concluded the effect of ouabain was not directly related to desensitization of the ACh receptor. It can be concluded from the article that the magnitude of the effects of ouabain on *Helix* may be related to an increase in the level of cAMP and, respectively, phosphorylation of the ACh receptor in neurons of type A, and the absence of phosphorylation of the receptor in type B neurons.

In a subsequent study, Grigorian et al. (2001) found ouabain-sensitive A-type muscarinic receptors and ouabain-insensitive B-type nicotinic receptors on the same neuron in *H. pomatia*. The activity of either A- or B-type receptor could depend on the physiological state of the neuron that could in turn depend on the phosphorylation state of the receptor and/or the level of activity of an endogenous ouabain-like compound.

Two cerebral cortex soluble fractions, named peaks I and II, which respectively stimulate and inhibit neuronal Na^+^/K^+^-ATPase activity, have been isolated by gel filtration in Sephadex G-50 (Rodriguez De Lores Arnaiz et al. 1997, 1998, 1999). Since earlier studies suggested a correlation between cholinergic transmission and Na^+^/K^+^-ATPase activity, Rodriguez De Lores Arnaiz et al. (1999) tested the effects of these peaks on binding of the muscarinic antagonist [^3^H]quinuclidinyl benzilate to these membranes. The authors found that binding was increased by peak I and decreased by peak II, II-E (a purified fraction of II) and by ouabain, these effects being concentration dependent. These results are similar to those found using synaptosomal membrane Na^+^/K^+^-ATPase, and so the authors concluded both systems functioned in a similar manner. Stimulation of the pump activates nicotinic and muscarinic cholinergic receptors, and the inhibition of the activity of this enzyme causes the opposite effect.

This conclusion supports the idea that endogenous modulators of the Na^+^/K^+^-pump exist and may physiologically regulate the pump that may indirectly define signalling by modulating other neurotransmitter receptors.

Studies involving pharyngeal and body wall muscle of *C. elegans* and the Na^+^/K^+^-pump are also included in this section since both muscles receive cholinergic innervation (Chiang et al. 2006; Rand et al. 2000; Richmond and Jorgensen 1999). *eat*-*6* encodes an orthologue of the *α* subunit of Na^+^/K^+^-ATPase in *C. elegans* (Davis et al. 1995). The properties of pharyngeal contractions of *eat*-*6* mutants differ from wild type in that they are weaker, slower and their relaxation delayed. Intracellular recordings from terminal bulb muscle fibres of *eat*-*6* mutants show that the MP is consistently depolarized and the action potentials (APs) reduced in amplitude. Davis et al. propose that due to reduced Na^+^/K^+^-pump activity, the ion gradients across the muscle fibres are reduced. Following ablation of the pharyngeal nervous system, the *eat*-*6* phenotype persists, suggesting that EAT-6 has a site of action in the muscle fibres. Interestingly, application of 20 µM ouabain to wild-type *C. elegans* dissected pharynxes caused a large reduction in the relaxation R transient of the electropharyngeogram (EPG). These EPGs are similar to those obtained from *eat*-*6* mutants. This effect of ouabain could be reversed following washing. Higher concentrations of ouabain (35–40 µM) caused muscles to hypercontract, an effect also observed in *eat*-*6* mutants.

These studies using *eat*-*6* mutants have been extended by Doi and Iwasaki (2008) who found that mutations in EAT-6 affected ACh synaptic efficacy by altering expression and localization of nAChRs at the *C. elegans* neuromuscular junction. It is proposed that the Na^+^/K^+^-pump may have a novel role like a scaffolding protein to help establish a rigid receptor cluster just beneath the presynaptic release site. These effects of EAT-6 Na^+^/K^+^-ATPase regulate cholinergic synaptic transmission independently of pump activity. Doi and Iwasaki also investigated the localization of the Na^+^/K^+^-ATPase *β* subunit, NKB-1, the most widely expressed of the three NKB *β* subunits in *C. elegans*. NKB-1 protein physically binds to EAT-6, and *nkb*-*1* mutants showed deficits similar to *eat*-*6* mutants, including defects in pumping. This suggests that EAT-6 and NKB-1 form a functional Na^+^/K^+^-ATPase in vivo. Doi and Iwasaki discuss possible mechanisms whereby Na^+^/K^+^-ATPase could induce nAChR clustering. For example, Na^+^/K^+^-ATPase may modulate nAChR trafficking through activation/inactivation of Src tyrosine kinase. It has been shown that binding of Src to Na^+^/K^+^-ATPase can form a functional signalling complex (Tian et al. 2006). It is also possible the number of postsynaptic cholinergic receptors of *eat*-*6* mutants could be increased. Doi and Iwasaki (2008) also found that levamisole and nicotine receptors of *eat*-*6* mutants were differentially affected in their expression and localization in the body wall muscle junction. The sensitivity to ACh agonists was also increased in *eat*-*6* mutants.

Ethanol can cause hypercontraction of *C. elegans* through activation of a novel *α* subunit associated with the cholinergic body wall muscle receptor (Hawkins et al. 2015). This hypercontraction can reverse after 40 min despite the continual presence of ethanol, indicating ethanol tolerance. The authors established a link between this cholinergic signalling, Na^+^/K^+^-ATPase and ethanol tolerance. For example, an unusual mutation in EAT-6, *eat*-*6* (*eg200*), failed to develop tolerance to ethanol-induced hypercontraction which suggest that Na^+^/K^+^-ATPase function is required for the development of ethanol tolerance in *C. elegans*.

*Glutamate receptors* Glutamate is the main excitatory synaptic transmitter in the mammalian brain and acts as a transmitter in invertebrates (Walker et al. 1996). In the 1990s, thanks to the use of molecular biological methods for studying glutamate receptors, they were divided into ionotropic (iGlu) and metabotropic (mGlu) (Mosharova 2001). NMDA, AMPA and kainate receptors are referred to as ionotropic (i.e., ion channel) receptors. All the other receptors are termed metabotropic (mGluRs) and regulate ion channels and enzymes that produce second messengers via specific receptors coupled to G-proteins. There are eight mGluRs, divided into 3 groups, I, II and III, depending on the level of conservation of their amino acid sequences and mode of action (Pin and Duvoison 1995). AMPARs mediate the vast majority of fast excitatory synaptic transmission (Trussell et al. 1994), whereas NMDARs play a vital role in the modulation of synaptic efficacy, generating synaptic plasticity (Hunt and Castillo 2012).

AMPARs are heterotetramers, assembled from different combinations of four subunits GluA1-4, the most common of which are receptors containing GluA1/GluA2 or GluAR2/GluA3. NMDARs are composed of GluN1 subunits, and at least one GluN2 subunit, out of four GluN2 subtypes, GluN2A-2D. AMPAR and NMDAR co-localize at the postsynaptic domain at a high density, probably stabilized and regulated, by interaction with cytosolic scaffolding proteins (Traynelis et al. 2010).

AMPARs are primarily sodium channels. In comparison, NMDARs allow entry of both sodium and calcium, with the latter playing an important role in synaptic plasticity, as calcium triggers a variety of downstream signalling events.

NMDARs play an important role in excitatory transmission, plasticity and excitotoxicity in the brain (Zhang et al. 2012a). Their activation enhances long-term potentiation and reduces long-term depression at Schaffer collateral-CA1 synapses in hippocampus. The NMDA receptor is simultaneously a potential-dependent and ligand-dependent ion channel that selectively transmits positively charged ions. The major part of the ion current consists of calcium and sodium ions that pass into the cell, releasing potassium ions from the cell. The NMDA receptor consists of four subunits, two NR1 class, and two NR2 class. A third NMDA receptor subunit, NR3, was subsequently identified and has been reviewed by Low and Wee (2010).

An endogenous Na^+^/K^+^-ATPase inhibitor, endobain E (fraction IIE), has been isolated from rat brain and shares several properties with ouabain. Endobain possesses neurotoxic properties attributable to Na^+^/K^+^-ATPase inhibition, which leads to NMDAR activation, supporting the concept that intracellular concentrations of Na^+^ and K^+^ ions may modulate NMDAR function (Reines et al. 2001, 2004). The effect of endobain E on expression of NMDA receptor subunits in membranes of rat cerebral cortex and hippocampus was analysed by Western blot (Bersier et al. 2008). Two days after administration of 10 µl endobain (1 μl per 28 mg tissue), NR1 subunit expression increased fivefold and 2.5-fold, respectively, in cerebral cortex and hippocampus. NR2A, NR2B and NR2D subunit expression increased in both brain areas. NR2C subunit expression was unaffected in either area. These results indicate that endobain E differentially modifies NMDA receptor subunit expression.

Excitatory synaptic transmission in the mammalian cortex involves activation of AMPARs and the entry of Na^+^ into the cell that has to be removed via activation of Na^+^/K^+^-ATPase. It is reasonable to assume the existence of crosstalk between these receptors and Na^+^/K^+^-ATPase. Interestingly, it has been shown that Na^+^/K^+^-ATPase is abundant at synaptic sites and is co-localized with AMPARs (Zhang et al. 2009). These authors propose an interaction between Na^+^/K^+^-ATPase *α*1 subunit and the intracellular C-terminal of GluR2 subunits. Following Na^+^/K^+^-ATPase inhibition, there is a rapid internalization and proteasomal-mediated degradation of AMPARs and suppression of AMPA-mediated synaptic transmission. This suggests a homeostatic regulation of AMPARs by Na^+^/K^+^-ATPase. It is proposed that the intracellular Na^+^ accumulation caused by Na^+^/K^+^-ATPase inactivity leads to a removal of Na^+^ channels in the cell surface. Ouabain-induced AMPAR degradation is abolished in the presence of proteasome inhibitors. It is possible that this degradation route is modulated by endogenous Na^+^/K^+^-ATPase inhibitors. In this way, the pump might play an important function to regulate AMPAR synaptic distribution and transmission that are essential components of plasticity (Man 2012). These can include endogenous ouabain, endobain and agrin (Hilgenberg et al. 2006; Schoner 2000, 2002). Thus, Na^+^/K^+^-ATPase can regulate AMPAR turnover, synaptic strength and brain function. Na^+^/K^+^-ATPase dysfunction following hypoxia, ischaemia and stroke is a major early pathological response (Zhang et al. 2009).

Na^+^/K^+^-ATPase and NMDARs play important roles in the regulation of learning and memory in the hippocampus (Zhang et al. 2012a), with the former acting as an ion transporter and the latter as ion channels. These authors used dihydro-ouabain to investigate its effects on NMDA currents in rat hippocampal CA1 neurons. Dihydro-ouabain (10–1000 µM) increased these NMDA currents but not through activation of protein kinase A or C. However, selective inhibitors of Src tyrosine kinase and mitogen-activated protein kinases (MARK) cascade did block dihydro-ouabain-induced NMDA currents. Zhang et al. (2012a) concluded that Src mediates the crosstalk between Na^+^/K^+^-ATPase and NMDARs to transduce the signals from Na^+^/K^+^-ATPase to the MARK cascade.

Activation of NMDA receptors changes intracellular concentrations of Na^+^ and K^+^, which are subsequently restored by Na^+^/K^+^-ATPase. It was observed that NMDA receptor and Na^+^/K^+^-ATPase interact with each other and this interaction was shown for both isoforms of *α* subunit (*α*1 and *α*3) of Na^+^/K^+^-ATPase expressed in neurons (Akkuratov et al. 2015). Using Western blotting, these authors showed that long-term exposure of the primary culture of rat cerebellar neurons to nanomolar concentrations of ouabain leads to a decrease in the levels of NMDAR subunits NR1 and NR2B that is probably mediated by the *α*3 subunit of Na^+^/K^+^-ATPase. This differs from earlier work where endobain E injection resulted in increased NMDAR expression in cerebral cortex and hippocampus (Bersier et al. 2008). The authors speculate this difference could be due to a difference in brain region or a difference between the mode of action of endobain E and ouabain. A decrease in enzymatic activity of the *α*1 subunit of Na^+^/K^+^-ATPase was also observed following NMDAR activation. This effect is mediated by an increase in intracellular Ca^2+^. Thus, Na^+^/K^+^-ATPase and NMDAR can interact functionally by forming a macromolecular complex which can be important for restoring ionic balance after neuronal excitation (Akkuratov et al. 2015). In addition, NMDAR function can be regulated by endogenous ouabain-like compounds.

*Toxicity effects of ouabain* Cellular Na^+^/K^+^-ATPase inactivation in neuro-glial cell cultures of cerebellum by 1 mM ouabain leads to glutamate (Glu) accumulation, hyperstimulation of glutamate receptors, higher Ca^2+^ and Na^+^ influxes into the cells through Glu-activated channels (Stelmashook et al. 1999). This process leads to cell swelling, mitochondrial de-energization and death of granule cells. However, addition of an NMDAR antagonist with ouabain prevented these responses. The authors suggest that a fall in Na^+^/K^+^-ATPase activity in neurons may contribute to the onset of chronic neurological disorders.

Several alpha-isoforms of Na^+^/K^+^-ATPase, possessing different sensitivity to ouabain, may have different signalling functions. Inhibition of rat neuronal Na^+^/K^+^-ATPase alpha-3 isoform at low (100 nM) ouabain concentration led to activation of MAP kinase cascade via PKC and PIP_3_ kinase. In contrast to ouabain-sensitive alpha3 isoform of Na^+^/K^+^-ATPase, a ouabain-resistant alpha1 isoform (inhibition with 1 mM of ouabain) of Na^+^/K^+^-ATPase regulates MAP kinase via Src kinase-dependent reactions. Using an Annexin V-FITC apoptotic test to determine the cells with early apoptotic features allows us to conclude that alpha3 isoform stimulates and alpha1 suppresses apoptotic process in cerebellum neurons. These data are the first demonstration showing participation of ouabain-resistant (alpha-1) and ouabain-sensitive (alpha-3) Na^+^/K^+^-ATPase isoforms in diverse signalling pathways in neuronal cells (Karpova et al. 2010a, b).

*Glutamate receptors in invertebrates* The important role of glutamatergic transmission across invertebrate classes highlights a route by which Na^+^/K^+^-pump might impact on glutamatergic transmission. This transmitter plays an important role at the NMJ of arthropods. Furthermore, it is a key determinant in the central nervous system in other major invertebrate classes (Walker et al. 1996). These important roles involve both homologous ionotropic and metabotropic glutamate receptors. There is a close relationship between glutamate receptors and higher forms of behaviour across phyla (see review Robbins and Murphy 2006). Despite this, to date no invertebrate glutamate receptors have been described that are modulated by Na^+^/K^+^-pump. However, glutamate and non-NMDA glutamate agonists depolarize leech glial and Retzius cells, altering intracellular Na^+^ activity and inducing an after-hyperpolarization (Dorner et al. 1994). This after-hyperpolarization is blocked by 100 µM ouabain and when external sodium is partially replaced with lithium. These experiments show that direct effects of glutamate and non-NMDA glutamate agonists can activate a Na^+^/K^+^-pump.

*GABA receptors* Mammalian GABARs are classified into GABA_A_, GABA_B_ and GABA_C_ receptors (Olsen 2018). GABA_A_ and GABA_C_ receptors are ionotropic, while GABA_B_ receptors are metabotropic. There is relatively little literature on the interaction between Na^+^/K^+^-ATPase and GABARs. Studies using rat brain RNA injected into *Xenopus* oocytes showed the effect of ouabain on GABA receptors (Arvanov 1990; Arvanov and Usherwood 1991). Four to ten days post-injection, the oocytes responded to applications of 1–100 µM, GABA, L-kainate and L-glutamate. All three compounds evoked inward currents. In ouabain-containing saline, the responses to GABA, L-kainate and L-glutamate were increased by 80–120%, 20–30% and 20–40%, respectively, in both folliculated and defolliculated oocytes. The reversal potentials for these agonist-induced currents did not change in the presence of ouabain. 100 µM ouabain also increased oocyte weight and volume. The authors proposed that ouabain, by increasing oocyte volume, increases the area of oocyte membrane containing receptors that is accessible to exogenously applied agonists. This suggests the effect of ouabain is direct. The key role of Na^+^/K^+^-ATPase in modulating Cl^−^ fluxes (see above) means it is possible this effect might modulate this important class of inhibitory receptor.

*Dopamine receptors* (*DARs*) Na^+^/K^+^-ATPase is involved in the regulation of DARs. DARs can interact with a range of molecules collectively termed dopamine receptor interacting proteins, DRIPs, which not only regulate receptor signalling but contribute to receptor trafficking and stability and the formation of the DAR signalling complex in cells (Kabbani and Levenson 2007). Bertorello et al. (1990) provided evidence that dopamine, through a synergistic effect on D1 and D2 receptors, inhibits the Na^+^/K^+^-ATPase activity of isolated striatal neurons. This leads to a transient depolarization of the MP, with a rise in intracellular Na^+^. The mammalian DARs are divided into two families, viz, D1 and D2. The D1 family contains D1 and D5 subtypes that are coupled to the heterotrimeric G-protein G_S_ and positively regulate adenylyl cyclase activity. The D2 family, viz, D2, D3, D4, subtypes are coupled to inhibitory G_I/O_ proteins and reduce adenylyl cyclase activity. Dopamine and other catecholamines modulate Na^+^/K^+^-ATPase activity through two mechanisms, viz, a direct effect on the enzyme and secondly, on catecholamine receptors but involving PKC and PKA pathways. The latter activate Na^+^/K^+^-ATPase through stimulation of PKC and PKA pathways in specific tissues (Therien and Blostein 2000). Dopamine binding to neostriatal neuronal D1 DARs inhibits Na^+^/K^+^-ATPase activity, while dopamine binding to D2 DARs activates sodium channels, which increases intracellular sodium and activates Na^+^/K^+^-ATPase (Aizman et al. 2000). Using co-immunoprecipitation and mass spectrometry, it was shown that D1 and D2 DARs exist in a complex with Na^+^/K^+^-ATPase (Hazelwood et al. 2008). These authors conducted biological assays with Na^+^/K^+^-ATPase and DARs co-expressed in HEK293T cells to investigate the impact of Na^+^/K^+^-ATPase on DAR function. Transfection of either D1 or D2 DARs into HEK293T cells resulted in a marked decrease in Na^+^/K^+^-ATPase *α*1 activity without a change in enzyme protein levels. DARs are able to reduce Na^+^/K^+^-ATPase function in the absence of dopamine and without a change in enzyme levels. This provides further evidence to support the importance of an interacting complex. Co-expression of the two proteins in a signalplex (a term to describe a receptor complex, consisting of a variety of protein interactions, see Hazelwood et al. 2010) resulted in reciprocal dampening of each other’s function. This work shows that interaction between DARs and *α*1 subunit of Na^+^/K^+^-ATPase results in reciprocal modulation of function between the two proteins, in both the presence and absence of ligands, providing a novel control mechanism for DAR signalling and ion balance in the cell.

DARs are involved in the adaptation of mouse striatal Na^+^/K^+^-ATPase activity following activation of opioid receptors by morphine (Wu et al. 2007). In vivo short-term morphine treatment stimulated Na^+^/K^+^-ATPase activity and this stimulation was inhibited by a D2R antagonist, while long-term morphine treatment inhibits Na^+^/K^+^-ATPase and this inhibition was inhibited by a D1R antagonist. A cAMP-dependent protein kinase A was involved in regulating Na^+^/K^+^-ATPase activity by morphine.

*Invertebrate DAR interaction with Na*^*+*^*/K*^*+*^*-ATPase* The complexity of dopamine signalling in the invertebrates is well established, and all the major phyla express homologues of mammalian DARS (Walker et al. 1996; Troppmann et al. 2014). An example is the interaction between Na^+^/K^+^-ATPase and dopamine receptors of acinar cells of the tick, *Ixodes scapularis* (Kim et al. 2016). Dopamine-induced salivary gland secretion was inhibited by ouabain (10 µM), which blocked fluid transport in type III acini. Kim et al. (2016) suggest that the target of the D1 receptor-mediated intracellular signalling for salivary gland secretion includes Na^+^/K^+^-ATPase. The basis of this functional interaction remains to be elucidated. Does it work by the kind of direct protein interactions described for mammals or by shifting the ionic gradients that are required for normal cell function? Evidence for such interactions in the invertebrate nervous system is lacking.

*Serotonin* (*5-hydroxytryptamine*) *receptors* (*5-HTRs*) 5-HTRs are classified into G-protein-coupled receptors (GPCRs) and ligand-gated ion channels and mediate both excitatory and inhibitory effects (Hoyer et al. 1994). There are at least six GPCRs, viz, 5-HT_1_, 5-HT_2_, 5-HT_4–7_, which can be divided into subtypes and one ligand-gated Na^+^ and K^+^ cation channel, 5-HT_3_. 5-HT modulates Na^+^/K^+^-ATPase activity in CA1 pyramidal neurons in rat hippocampus (Zhang et al. 2012b). This inhibition is through 5-HT_3_Rs as it was reduced by a 5-HT_3_R antagonist but not by a 5-HT_1_R antagonist. In addition, a 5-HT_3_R agonist mimicked the effect of 5-HT. 5-HT agonists can modify Na^+^/K^+^-ATPase activity in the cerebral cortex of rats from day 21 onwards (Hernández 1982), and this effect is blocked by 5-HT antagonists. Following a state of induced 5-HT receptor hypersensitivity, the response of Na^+^/K^+^-ATPase to 5-HT agonists was enhanced. The author concluded that 5-HT receptor sensitivity in the rat brain involves Na^+^/K^+^-ATPase.

5-HT acts as a transmitter in all the major phyla (Walker et al. 1996). However, there is little evidence for interactions between 5-HTRs and the Na^+^/K^+^-pump. Injection of Na^+^ into leech, *H. medicinalis*, T sensory neurons results in the MP becoming more negative due to activation of Na^+^/K^+^-ATPase (Catarsi and Brunelli 1991). This increase in negativity is blocked by 5-HT which directly inhibits Na^+^/K^+^-pump activity in T cells through cAMP (Catarsi et al. 1993). Repetitive stimulation of T cell receptive field induces an enhanced after-hyperpolarization (AHP) in T cells which is mainly due to increased Na^+^/K^+^-ATPase activity (Scuri et al. 2002). The AHP is reduced by 5-HT or inhibition of the Na^+^/K^+^-pump that may facilitate action potential conduction in synaptic terminals and be important for short-term plasticity (Scuri et al. 2007). Inhibition of the Na^+^/K^+^-pump, following injection of 10 nM dihydro-ouabain, results in a more rapid swimming behaviour, suggesting a role for the pump in the physiology of swimming in the leech. However, the interaction between 5-HT and the Na^+^/K^+^ pump at the molecular level in the leech is not known.

## Possible intracellular mechanisms that are responsible for the control of neurotransmitter receptors by the Na^+^/K^+^-ATPase

There are several ways to regulate the activity of the transmitter receptors of the membrane with a Na^+^/K^+^-pump. First, when the volume of a neuron changes, the number of functionally active membrane chemoreceptors changes (Ayrapetyan 1990; Suleimanyan 1990).

On the other hand, inactivation of the Na^+^/K^+^-pump results in an increase in the level of ATP and cAMP (Arvanov et al. 1992b; Saghian et al. 1996), reduction in the level of cGMP and stimulation of the phosphorylation of the neuron membrane of *Helix* (Ayrapetyan 1990). These authors concluded there was a close correlation between Na^+^/K^+^-ATPase activity, the intracellular level of cyclic nucleotides and the level of membrane phosphorylation.

Inactivation of the Na^+^/K^+^-pump by ouabain results in suppression of ligand binding to receptors, as well as a decrease in the affinity of membrane chemoreceptors to the corresponding neurotransmitters (Arvanov 1990). The inhibitory effect of ouabain on the ACh responses of the neuronal membrane is a Ca^2+^-dependent process. Thus, the action of ouabain on neuronal responses of type A *Helix* neurons was eliminated by the addition of 10^−4^ M EGTA to the intracellular medium, that is, by binding intracellular free Ca^2+^. Similar results on the Ca^2+^ dependence of the inhibitory effect of ouabain on somatic cholinergic receptors of *Helix* neurons were obtained by Pivovarov and Boguslavskii (2001).

## Na^+^/K^+^-pump and regulation of neurotransmitter receptors by endogenous neuropeptides

In the mammalian central nervous system, neuropeptides can act directly via specific receptors to modulate synaptic activity or indirectly through the release of a classical transmitter. For example, the inhibitory effect of oxytocin is through the release of GABA (Huber et al. 2005), while vasopressin reduces the excitatory effect of glutamate by reducing glutamate release (Bailey et al. 2006). Other neuropeptides that enhance or reduce glutamate or GABA release include neuropeptide Y, somatostatin, dynorphin, hypocretin, glucagon-like peptide 1 and leptin (for review, van den Pol 2012). However, there is no evidence these effects involve Na^+^/K^+^-ATPase although interactions between neuropeptides and Na^+^/K^+^-ATPase have been demonstrated. For example, neurotensin inhibits Na^+^/K^+^-ATPase activity of synaptosomal membranes from rat cerebral cortex (Lopez Ordieres and Rodriguez de Lores Arnaiz 2000). Neurotensin exerts these effects through activation of NTS1 receptors that results in PI hydrolysis (Pereyra-Alfonso et al. 2008). In contrast, sub-µM angiotensin-(1-7) activates Na^+^/K^+^-ATPase from rat cerebral cortex synaptosomal membranes (Lopez Ordieres et al. 1998). *β*-Amyloid has also been proposed as a regulator of Na^+^/K^+^-ATPase (Petrushanko et al. 2016). These authors found that *β*-amyloid inhibited Na^+^/K^+^-ATPase activity of neuroblastoma cells by binding between the *α* and *β* subunits of the enzyme and preventing the subunits moving towards each other. Prolonged disruption of neuronal function enhanced the onset of dementia.

**Fig. 3:**
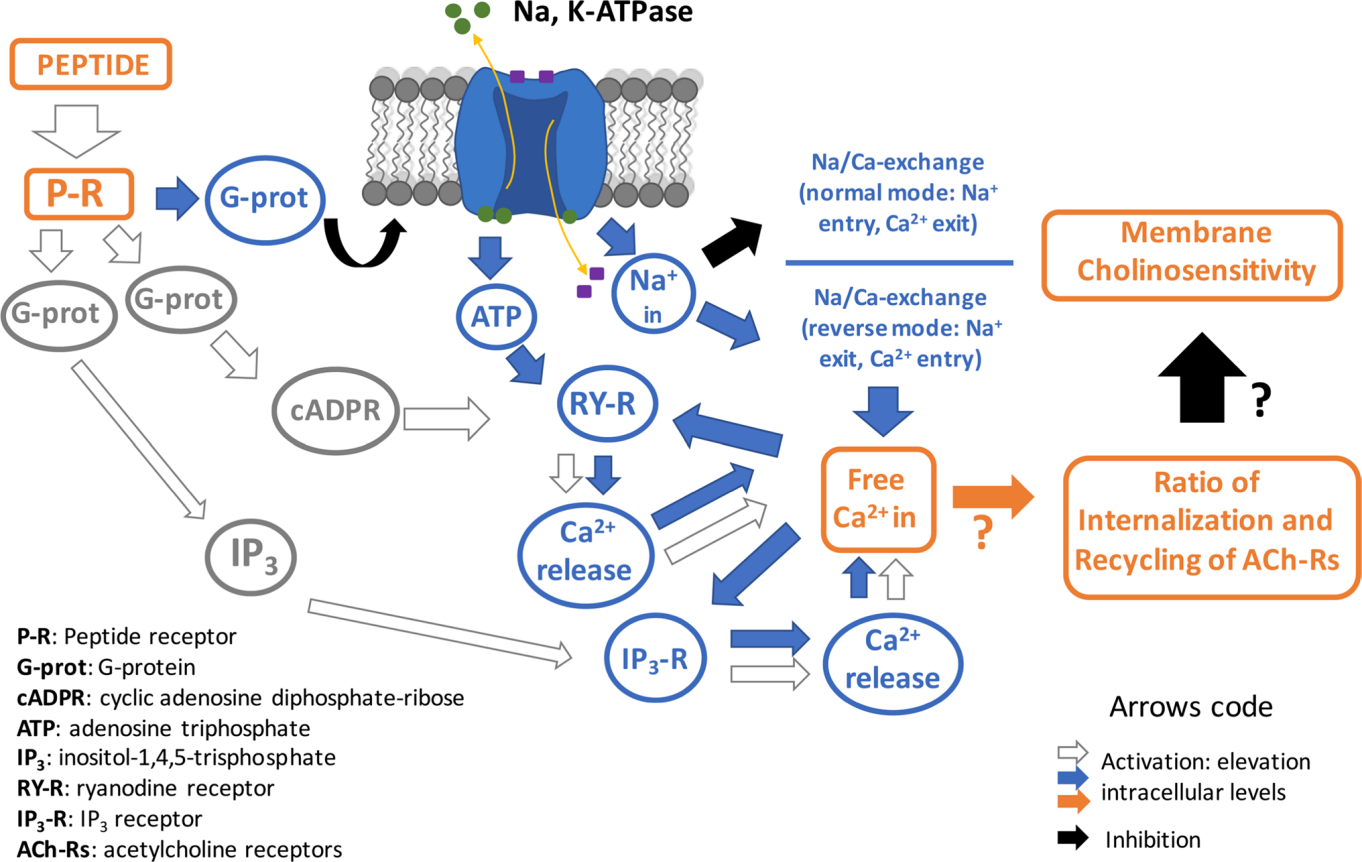
A diagram to summarize a putative intracellular mechanism for a peptide-mediated reduction of neuron sensitivity to a chemical transmitter, for example, acetylcholine. P-R, peptide receptor; G-prot, G-protein; cADPR, cyclic adenosine diphosphate-ribose; ATP, adenosine triphosphate; IP_3_, inositol-1,4,5-triphosphate; RY-R, ryanodine receptor; IP_3_-R, IP_3_ receptor; ACh-R, acetylcholine receptor; activation/increase of intracellular level, white/blue/orange arrow; inhibition of intracellular level, black arrow Modified from Pivovarov et al. (2007)

There is considerable literature on neuropeptide modulation of invertebrate neural circuits that in turn regulate many animal behavioural and physiological processes (for review, Taghert and Nitabach 2012). However, Na^+^/K^+^-ATPase has not been reported to play a role in these processes, but it has been proposed that the *α* subunit of the enzyme is an important regulator of the clock-controlled plasticity of the brain of *D. melanogaster* (Damulewicz et al. 2013). The rhythmic abundance of Na^+^/K^+^-ATPase in the optic lamina of *D. melanogaster* depends on CRY (a blue-light-sensitive protein encoded by the cryptochrome [*cry*] gene) and on peptidergic clock neurons producing the neuropeptide, Pigment Dispersing Factor and the Ion Transport Peptide. The best evidence for endogenous neuropeptides modulating classical transmitter function through Na^+^/K^+^-ATPase comes from studies using *Helix* neurons. For example, on *Helix* neurons, similar effects of ouabain and a number of endogenous peptides of the FMRFamide family have been observed on the amplitude of ACh-induced currents (Arvanov and Ayrapetyan 1980; Pivovarov and Walker 1996). SEPYLRFamide and a number of similar FMRFamide-like peptides act as inhibitory modulators of ACh receptors of *H. lucorum* neurons. Ouabain (0.1 mM, bath application) decreased the ACh-induced inward current (ACh current) and increased the leak current. Results indicated that Na^+^/K^+^-pump was responsible for the SEPYLRFamide-mediated inhibition of ACh receptors in *Helix* neurons. Na^+^/Ca^2+^-exchange and intracellular Ca^2+^ released from internal pools containing TG-sensitive Ca^2+^-pump are involved in the Na^+^/K^+^-pump pathway for the SEPYLRFamide-mediated inhibition of ACh receptors (Pivovarov et al. 2007). The evidence for these inhibitory events being linked is as follows: (1) ouabain decreased the modulatory SEPYLRFamide effect on the ACh current; (2) there was a correlation between the effects of ouabain on the amplitude of the ACh current and on the modulatory peptide effect; (3) ouabain and SEPYLRFamide inhibited the activity of *H. aspersa* brain Na^+^/K^+^-ATPase; (4) activation of Na^+^/K^+^-ATPase by intracellular injection of 3 M Na acetate or 3 M NaCl reduced the modulatory peptide effect on the ACh current. An inhibitor of Na^+^/Ca^2+^ exchange, benzamil (25 µM, bath application), and an inhibitor of Ca^2+^-pump in the endoplasmic reticulum, thapsigarin (TG, applied intracellularly), both prevented the effect of ouabain on SEPYLRFamide-mediated modulatory effect. Another inhibitor of Ca^2+^-pump in the endoplasmic reticulum, cyclopiazonic acid (applied intracellularly) did not prevent the effect of ouabain on SEPYLRFamide-mediated modulatory effect. These results indicate that Na^+^/K^+^-pump is responsible for the SEPYLRFamide-mediated inhibition of ACh receptors in *Helix* neurons. Na^+^/Ca^2+^ exchange and intracellular Ca^2+^ released from internal pools containing TG-sensitive Ca^2+^-pump are involved in the Na^+^/K^+^-pump pathway for the SEPYLRFamide-mediated inhibition of ACh receptors (Pivovarov et al. 2007). These results are summarized in Fig. 3.

In another invertebrate preparation, myomodulin-induced inhibition of Na^+^/K^+^-pump in heart oscillator interneurons of the leech, *H. medicinalis*, speeds up bursting of these neurons, an effect mimicked by ouabain (Tobin and Calabrese 2005). Myomodulin also potentiated the hyperpolarization-activating cation current, I_h_, of these neurons.

## Conclusion

The work presented in this review provides evidence that the Na^+^/K^+^-pump not only functions as a cationic Na^+^/K^+^ antiporter-like molecule, but also as a regulatory system involved in controlling membrane neurotransmitter receptor function.

Na^+^/K^+^-ATPase forms functional complexes in which the enzyme and the transmitter receptor influences each other’s actions in the cell, resulting in the modification of cell signalling and subsequent physiology of the organism. The enzyme is also involved in monitoring the effectiveness of membrane receptors (Boldyrev et al. 2004; Krivoi et al. 2006; Hazelwood et al. 2008; Man 2012). In neurons, the influence of the Na^+^/K^+^-pump on postsynaptic receptors provides control over the efficiency of synaptic transmission in the brain. The latter is involved in higher cognitive functions—neuronal plasticity, learning and memory (Gardoni et al. 2009; Zhang et al. 2012a).

While many of the examples of Na^+^/K^+^-pump-transmitter receptor interactions in this review are from studies using mammalian preparations, it is likely that all transmitter receptors in invertebrates can also be influenced by Na^+^/K^+^-pump modulation. It is also very likely that endogenous ouabain-like molecules exist throughout the invertebrates and modulate transmitter function through their modulation of transmitter receptors and influence on pathophysiology. These are areas for future research using invertebrate preparations.
